# Glances at the Hospitals

**Published:** 1904-09-24

**Authors:** 


### GLANCES AT THE HOSPITALS.

### THE ISLE OF WIGHT HOSPITAL.

THE ordinary income received during the year was £3,232, and the expenditure was £3,492, showing a deficit of £260. At the beginning of the year there were 52 patients in residence, and 477 were admitted, making a total of 529 under treatment. Of these 263 were discharged cured, 158 discharged relieved, 33 for other reasons, 32 died, and 43 remained in the wards. The average number of beds occupied was 43, and the average duration of treatment was 27 days. We did not notice any statement relative to the cost per bed occupied or the cost per patient. The medical and surgical tables are prepared in such a manner as to be quite useless for purposes of comparison; 318 operations were performed during the year, but no anæsthetic table is given.

### NORFOLK AND NORWICH HOSPITAL.

THE actual income was £12,511, and the actual expenditure was £12,666. There is a bank overdraft of £2,524. On the first of January the hospital contained 164 patients; 1,825 were admitted during the year, making a total of 1,989 under treatment, showing an increase of 170 over the previous year. The daily average number resident was 169, and the average stay in the hospital was 30 days. The cost per bed occupied was £65 13s., and the average cost per patient was £1 5s. 3d. a week. The medical and surgical statistics are drawn up with much care, and give information on all essential points. There were 16 cases of acute rheumatism, and of these 15 were discharged cured, and 1 remained under treatment. Of 12 cases of enteric fever, 8 were cured, 1 died, and 3 remained in the wards. Of 13 cases of pneumonia, 8 were cured, 4 died, and 1 remained under treatment. Eight hundred surgical operations were performed, but here no results are given, nor is there any table showing the nature of the anæsthetic used—omissions which we regret to notice in an otherwise good report.

